# Shelter Hospital: Glimmers of Hope in Treating Coronavirus 2019

**DOI:** 10.1017/dmp.2020.105

**Published:** 2020-04-16

**Authors:** Feng Zhou, Xuan Gao, Mengwei Li, Ying Zhang

**Affiliations:** Department of Endocrinology, Puren Hospital of Wuhan University of Science and Technology, Wuhan, China; Department of Critical Care Medicine, Zhongnan Hospital of Wuhan University, Wuhan, China

**Keywords:** COVID-19, shelter hospital, infection control

## Abstract

Since the first report of the 2019 novel coronavirus disease (COVID-19) in December 2019 in Wuhan, China, the outbreak of the disease has been continuously evolving. Until March 17, 2020, 185, 178 cases had been confirmed, including 81,134 cases in China and 104,044 cases outside of China. In this comment, we report the unexpected beneficial effect of a deployable rapid-assembly shelter hospital on the prevention and treatment of COVID-19. We describe the shelter hospital maintenance, treatment mode and primary treatment methods, which will provide a valuable experience in dealing with public health emergencies, such as COVID-19, for other countries and areas.

In December 2019, the type of pneumonia caused by the 2019 novel coronavirus disease (COVID-19) occurred in Wuhan, Hubei province, China,^1,2^ spreading rapidly to other parts of Hubei province and China.^1,2^ Since the middle of January 2020, the number of patients affected with COVID-19 has increased rapidly from 100 cases per day to more than 1000 cases per day.^3^ On February 2, the total number of patients diagnosed with COVID-19 was 5142 in Wuhan; however, only 1000 hospital beds were available for the treatment of COVID-19.

A large number of patients crowded into fever clinics and could not receive treatment in time. Many medical staff became infected when they were in contact with patients with COVID-19, leading to the shortage of medical staff and making the treatment of COVID-19 more inefficient. Patients, their family members, and uninfected residents experienced worry, anxiety, fear, and panic. Although the 2 newly built hospitals would soon be put into use, obviously, they could not meet the needs of accommodating patients with COVID-19.

Under these circumstances, the Chinese Government decided to build a deployable rapid-assembly shelter hospital for the treatment of mild COVID-19 patients. Three large-space, multi-bed shelter hospitals were built at breakneck speed in Hongshan Gymnasium (800 beds), Wuhan Living Room (2000 beds), and Wuhan International Convention and Exhibition Center (1000 beds) on the evening of February 3, 2020. On the following day, 8 shelter hospitals were constructed in large indoor exhibition centers, stadiums, or schools in urban areas such as Hanyang, Jiangan, Qiaokou, Hongshan, Jiangxia, and Huangpi Districts.

Based on the medical shelter model used before,^4^ the shelter hospital was divided into medical, nursing, laboratory testing, and ward areas. Medical staff were selected from hospitals in Wuhan or other areas, comprising doctors, nurses, paramedics, physician’s assistants, X-ray technicians, respiratory therapists, and logistical personnel. Body temperature, blood oxygen saturation, and blood pressure were monitored 3 times a day. Necessary medications, such as antiviral therapy and aerosol inhalation, were prescribed. Psychological counseling was performed for the patients who were experiencing fear, anxiety, or panic. Square dancing and Tai Chi playing were performed 2 times a day. Nutritious and delicious diets were provided.

Patients would be transferred to a designated hospital for further treatment if their conditions became more severe. They would not be discharged until all clinical symptoms and CT imaging abnormalities had resolved, with the addition of 2 consecutive SARS-CoV-2 quantitative real-time reverse transcription polymerase chain reaction (qRT-PCR) test results of negative. After being discharged, patients were required to continue the quarantine protocol at a rehabilitation station for another 14 days.

As a prehospital diagnosis and treatment section, the shelter hospital played a significant role in improving the ability to treat COVID-19 and prevent its spread. Patients crowded in the fever clinic were transferred to shelter hospitals, which realized the source control of infection and cut off the means of transmission. The risk of infection of the patients’ family members was rapidly reduced, and the spread in the family community was effectively avoided. The workload of medical staff was reduced, and more hospital beds were available for severe and critically ill patients who needed to be hospitalized. Unnecessary public panic was avoided, and glimmers of hope finally appeared.

During the epidemic prevention, 16 shelter hospitals were built in Wuhan, providing 13 467 beds and treating more than 12 000 patients. All patients were treated properly and no death occurred. The number of new cases declined slowly from the peak, and the number of patients discharged from the shelter hospital continued to increase. On the afternoon of March 10, 2020, all patients in 16 shelter hospitals were discharged and all shelter hospitals were declared closed, representing a temporary victory of prevention and treatment of COVID-19 in Wuhan, China.

As of March 17, 2020, the locations with confirmed COVID-19 include 110 countries worldwide, and 185 178 cases were confirmed, including 81 134 cases in China and 104 044 cases outside of China.^5^ The 16 “Ark of Life" set sail at the most critical moment of the epidemic, with 94 medical teams and more than 8000 medical personnel becoming fearless sailors on the Ark. The deployable rapid-assembly shelter hospital is a major public health measure taken by the government of Wuhan, China, in this epidemic prevention and control campaign, and it has become a valuable experience in dealing with public health emergencies around the world.

## References

[1] Zhu N , Zhang D , Wang W , et al. A novel coronavirus from patients with pneumonia in China, 2019. N Engl J Med. 2020;382:727-733. 31978945 10.1056/NEJMoa2001017 PMC7092803

[2] Huang C , Wang Y , Li X , et al. Clinical features of patients infected with 2019 novel coronavirus in Wuhan, China. Lancet. 2020;395:497-506. 31986264 10.1016/S0140-6736(20)30183-5 PMC7159299

[3] Chan JF , Yuan S , Kok KH , et al. A familial cluster of pneumonia associated with the 2019 novel coronavirus indicating person-to-person transmission: a study of a family cluster. Lancet. 2020;395:514-523. 31986261 10.1016/S0140-6736(20)30154-9 PMC7159286

[4] Bai S , Yu BG , Zhang YZ , et al. Challenges of treating adenovirus infection: application of a deployable rapid-assembly shelter hospital. Disaster Med Public Health Prep. 2018;12:109-114. 28260561 10.1017/dmp.2016.187

[5] China National Health Commission. Update on pneumonia caused by the new coronavirus infections. Updated March 17, 2020. http://www.nhc.gov.cn/xcs/yqtb/202003/28d026a0422844969226913ee3d56d77.shtml. Accessed March 17, 2020.

